# Aplicabilidade de Algoritmos de *Machine Learning* no Diagnóstico de Fibrilação Atrial e SQTL por Interpretação de Eletrocardiograma: Uma Revisão Sistemática

**DOI:** 10.36660/abc.20240843

**Published:** 2025-08-12

**Authors:** Paulo Cainan Guimarães do Nascimento, Matthews Silva Martins, Alex Cleber Improta-Caria, Roque Aras

**Affiliations:** 1 Universidade Federal da Bahia Faculdade de Medicina da Bahia Salvador BA Brasil Universidade Federal da Bahia – Faculdade de Medicina da Bahia, Salvador, BA – Brasil; 2 Universidade Federal do Espírito Santo Laboratório de Fisiologia Molecular e Inteligência Artificial Departamento de Ciências Fisiológicas Vitória ES Brasil Universidade Federal do Espírito Santo – Laboratório de Fisiologia Molecular e Inteligência Artificial, Departamento de Ciências Fisiológicas, Vitória, ES – Brasil; 3 Universidade de São Paulo Laboratório de Bioquímica e Biologia Molecular do Exercício Escola de Educação Física e Esporte São Paulo SP Brasil Universidade de São Paulo – Laboratório de Bioquímica e Biologia Molecular do Exercício, Escola de Educação Física e Esporte, São Paulo, SP – Brasil

**Keywords:** Arritmias Cardíacas, Inteligência Artificial, Aprendizado de Máquina, Rede Nervosa, Eletrocardiografia

## Abstract

**Fundamento:**

*Machine learning* (ML) é um tipo de algoritmo que aprende de forma autônoma a reconhecer padrões complexos. No contexto diagnóstico de arritmias cardíacas, esses algoritmos demonstraram avanços significativos devido à sua capacidade de fornecer interpretação automatizada e reconhecimento de padrões em eletrocardiogramas (ECGs).

**Objetivo:**

Analisar e identificar a aplicabilidade, validade e viabilidade de modelos de algoritmos de ML no processo diagnóstico de arritmias cardíacas por meio de interpretação eletrocardiográfica automatizada.

**Métodos:**

Esta revisão sistemática da literatura foi relatada de acordo com as diretrizes PRISMA. As buscas foram realizadas na Biblioteca Cochrane, EMBASE, LILACS e PubMed entre fevereiro de 2022 e novembro de 2022. O período do estudo abrange artigos publicados entre 2017 e 2022.

**Resultados:**

A busca no banco de dados gerou 119 resultados, abrangendo três subtemas: Síndrome do QT Longo (SQTL), intervalo QT corrigido (QTc) e fibrilação atrial (FA). A FA foi o tema mais prevalente. Os tamanhos das amostras foram bastante variáveis. Os resultados foram, em sua maioria, satisfatórios. No diagnóstico de SQTL usando Inteligência Artificial (IA), o algoritmo superou os métodos convencionais na distinção diagnóstica. Na avaliação do QTc, não houve diferença entre o ECG integrado à IA e o ECG convencional. No diagnóstico de FA, os algoritmos, modelos e dispositivos demonstraram alta sensibilidade e especificidade, além de maior acurácia.

**Conclusão:**

Os modelos de ML no processo diagnóstico de arritmias cardíacas são viáveis e estão em rápido desenvolvimento. Apresentam valores de acurácia entre 96,4% e 98,2%, sensibilidade entre 92,8% e 99,4% e especificidade entre 95% e 98,1%, particularmente no diagnóstico de fibrilação atrial.

## Graphical abstract

**Figura Central:**
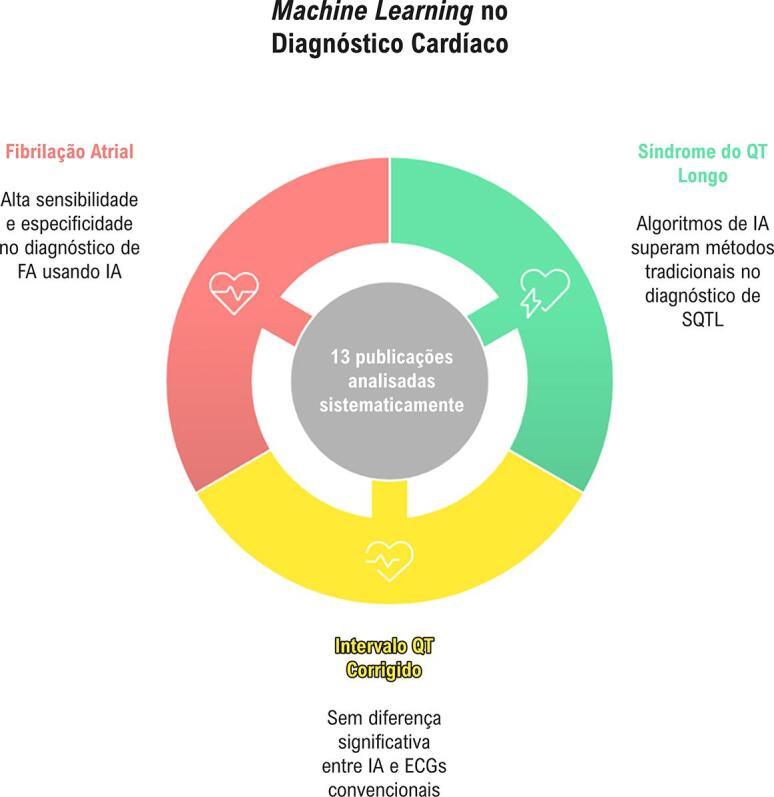
Aplicabilidade de Algoritmos de *Machine Learning* no Diagnóstico de Fibrilação Atrial e SQTL por Interpretação de Eletrocardiograma: Uma Revisão Sistemática

Figura criada usando Napkin.ai

## Introdução

Segundo a Organização Mundial da Saúde, as doenças cardiovasculares (DCV) são as principais causas de morte em todo o mundo, representando 32%.^1^ Entre as DCV, as arritmias são as mais comuns e se caracterizam por anormalidades na geração ou condução de impulsos elétricos, ocorrendo devido à função cardíaca anormal, resultando em ritmos cardíacos irregulares. A ocorrência de arritmias pode ser indolente, mas pode causar consequências graves.^2 , 3^

A abordagem diagnóstica para arritmias cardíacas envolve dois componentes principais: história clínica e eletrocardiograma (ECG). A história clínica lista batimentos cardíacos irregulares, dispneia, fadiga, tontura e, quando não tratada, histórico de acidente vascular cerebral e insuficiência cardíaca. A suspeita pode ser corroborada por alterações no exame físico cardiovascular por meio de ausculta com estetoscópio e palpação de pulsos. O diagnóstico é confirmado com o registro do ECG Holter por 24 horas e posterior certificação de ritmos irregulares por um médico especialista. Entretanto, o método convencional de diagnóstico por ECG Holter, em geral, impõe limitações devido à falta de flexibilidade mecânica ao paciente e ao método de análise retrospectiva, sem monitorização e acompanhamento em tempo real.^4 , 5^

No entanto, algoritmos de Inteligência Artificial (IA) e seus derivados surgiram como um método confiável para identificar e classificar padrões eletrocardiográficos que podem sugerir anormalidades.^5^ Dorado-Díaz et al.^6^ referem-se à IA como um “campo da ciência da computação que tenta imitar o processo cognitivo humano, a capacidade de aprendizagem e o armazenamento de conhecimento”. Em Ciências da Saúde, algoritmos de IA são predominantemente aplicados em predição, recomendação e suporte diagnóstico com base em modelos de *Machine learning* (ML) e *Deep Learning* (DL). Conceitualmente, o ML se concentra no desenvolvimento de algoritmos que permitem que sistemas de computador aprendam sem programação explícita. Em contraste, os modelos de DL derivam suas capacidades preditivas de Redes Neurais Artificiais (RNAs) com múltiplas camadas de processamento de informações.^7 , 8^

Nesse contexto, algoritmos de ML agora são capazes de analisar ECGs, fornecer interpretações automatizadas e prever riscos associados.^8^ A literatura destaca previsões derivadas da otimização de etapas processuais, como processamento de sinais, extração de variáveis significativas e classificação de algoritmos.^9^ Apesar da complexidade computacional intrínseca, há diversos avanços em sistemas que desempenham essa função.

Portanto, ferramentas de IA podem complementar o monitoramento médico, desde o nível preventivo até o de reabilitação. O desenvolvimento de ML capaz de conduzir análises de ECG é essencial na detecção precoce de condições cardíacas anormais remotamente e no auxílio ao processo diagnóstico de arritmias e outras condições de saúde.^5^ Além disso, o monitoramento cardíaco de longo prazo, o reconhecimento e a classificação de arritmias com base na análise do traçado de ECG podem ser um processo demorado e desnecessariamente exaustivo para o cardiologista. Assim, técnicas de detecção de arritmia autonômica são úteis para tais execuções. Nesse cenário, técnicas computadorizadas de reconhecimento de padrões potencialmente ajudarão a fornecer diagnóstico e intervenção médica sempre que necessário.^3^

Considerando o exposto, o estudo da IA e suas tecnologias derivadas na área da saúde se mostra altamente relevante. Portanto, este trabalho teve como objetivo, por meio de uma revisão sistemática da literatura científica, explorar a aplicabilidade, a validade e a viabilidade de modelos de ML no processo diagnóstico de arritmias cardíacas. O estudo buscou identificar os benefícios e as limitações metodológicas, os potenciais impactos clínicos e sociais dessas tecnologias e suas implicações para a estrutura da educação e da prática médica.

## Métodos

O delineamento do estudo é uma revisão sistemática da literatura conduzida utilizando os critérios estabelecidos pelo método PRISMA ( *Preferred Reporting Items for Systematic Reviews and Meta-Analyses* ), conforme detalhado nas diretrizes metodológicas para elaboração de revisões sistemáticas e metanálises de ensaios clínicos randomizados fornecidas pelo Ministério da Saúde do Brasil.^10^

Para a busca bibliográfica sobre o tema pretendido, foram utilizadas as seguintes bases de dados: Biblioteca Cochrane, Embase, LILACS (Literatura Latino-Americana e do Caribe em Ciências da Saúde) e PubMed-MEDLINE (Sistema Online de Análise e Recuperação de Literatura Médica). Os termos de busca foram derivados do vocabulário DeCS/MeSH (Descritores em Ciências da Saúde/Cabeçalhos de Assunto Médico), complementados por termos de entrada. Os descritores incluíram: arritmias; arritmia cardíaca ou cardíaca; *machine learning* ; *machine learning* não supervisionado; *machine learning* supervisionado; *deep learning* ; redes neurais, computador ou redes neurais de computador ou rede neural computacional ou redes neurais computacionais; inteligência artificial ou IA ou inteligência computacional; e eletrocardiografia ou ECG ou EKG. A busca foi realizada utilizando a seguinte configuração de descritores e operadores booleanos: *((cardiac arrhythmia) OR (arrhythmia) AND (machine learning)) OR (unsupervised machine learning)) OR (supervised machine learning)) OR (deep learning) OR (neural network) OR (artificial intelligence) AND (electrocardiography).*

Durante o processo de triagem, foram considerados os estudos que atenderam aos seguintes critérios de inclusão: 1) pesquisas envolvendo seres humanos ou dados obtidos de bancos de dados humanos; 2) publicações em sua versão final em periódicos científicos revisados por pares; 3) estudos dos seguintes tipos: estudos de caso-controle, estudos de coorte, ensaios clínicos, relatos de caso e séries de casos; 4) publicados entre 2017-2022; 5) disponíveis em português ou inglês. Os critérios de exclusão incluíram: 1) publicações em idiomas diferentes de português ou inglês; 2) estudos com descrições experimentais sem aplicação clínica do modelo algorítmico; 3) artigos não relacionados ao diagnóstico de arritmias cardíacas; 4) artigos que não aplicam modelos de IA ao diagnóstico de arritmias cardíacas; 5) artigos que propõem métodos para diagnóstico de arritmias cardíacas usando exames diferentes do ECG; 6) artigos que descrevem o uso de IA para diagnóstico de condições diferentes de arritmias cardíacas; 7) outros tipos de publicações, como editoriais, diretrizes, livros, revisões sistemáticas e meta-análises; 8) resultados duplicados nos bancos de dados pesquisados.

O processo de triagem dos resultados da busca e extração de dados foi baseado no método PRISMA e realizado por um único operador. Esse processo resultou na criação de um banco de dados e de um modelo de tabela, permitindo a organização dos dados recuperados em configurações alinhadas aos critérios do método empregado, servindo como precursor para análises futuras. Utilizando essa metodologia, o objetivo foi avaliar estudos clínicos que abordassem o uso e a aplicabilidade de algoritmos de ML no processo diagnóstico de arritmias cardíacas por meio da interpretação automatizada de ECGs.

## Resultados

**Figura 1:**
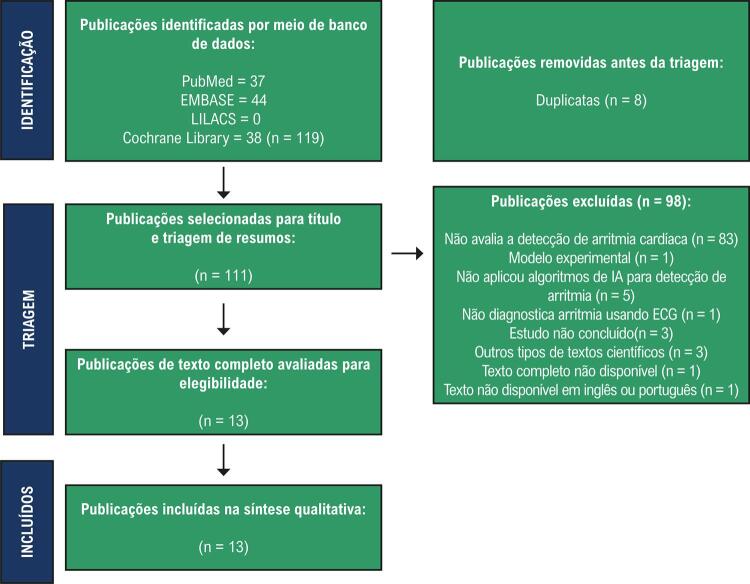
– Fluxograma da Revisão Sistemática da Literatura.

A busca nas bases de dados gerou um total de 119 resultados, incluindo 38 da Biblioteca Cochrane, 44 da Embase, nenhum da LILACS e 37 da PubMed. Após a aplicação dos critérios de exclusão, 98 publicações foram excluídas pelos seguintes motivos: não avaliaram a detecção de arritmias cardíacas (83), não aplicaram algoritmos de IA para detecção de arritmias (5), abordaram estudos em andamento (3), foram classificadas como outros tipos de textos científicos (3), focaram em um modelo experimental (1), descreveram a avaliação diagnóstica de arritmias usando métodos diferentes do ECG (1), não possuíam uma versão completa do texto (1) e não forneceram o texto completo em inglês ou português (1). Consequentemente, 13 publicações foram consideradas elegíveis para revisão completa e sistemática ( Figura 1 ).

Os 13 artigos considerados elegíveis foram incluídos na síntese qualitativa ( Tabela 1 ). A busca final foi realizada em 30 de novembro de 2022. Em relação aos países de origem das publicações incluídas, elas representavam sete locais: Estados Unidos (4), China (3), Coreia do Sul (2), Alemanha (1), França (1), Taiwan (1) e Finlândia (1). Em termos de delineamento do estudo, os artigos compreenderam estudos de coorte prospectivos (7), estudos de coorte retrospectivos (2), estudos de caso-controle (2) e ensaios clínicos randomizados (2). Todos os estudos receberam aprovação dos comitês de ética de suas respectivas instituições.

**Tabela 1:** – Síntese do Desenho, Resultados e Conclusões - SQTL

Autor, ano, (ref. Nº)	Bos, 2021^**11**^
Desenho do estudo	Estudo de caso-controle. Objetivo: Determinar se a IA é capaz de distinguir pacientes com SQTL de pacientes com QTc normal usando ECG de 12 derivações. Amostra: 2.984 pacientes previamente diagnosticados com SQTL ou que receberam alta como saudáveis. O diagnóstico de caso-controle foi realizado usando ECGs de 12 derivações disponíveis de 2.059 pacientes. Desfecho principal: A IA foi capaz de distinguir pacientes com SQTL, prolongamento persistente do intervalo QT e diferenciação genética.
Modelo IA	Rede Neural Profunda.
Resultados	A IA foi capaz de distinguir entre as duas populações (pacientes com SQTL e pacientes sem SQTL). Precisão: 82,2%; sensibilidade: 83,7%; especificidade: 80,6%; VPP: 83,2%; e VPN: 81,3%.
Conclusão	O ECG-IA superou o ECG convencional na distinção entre pacientes com SQTL e aqueles que receberam alta sem o diagnóstico. Além disso, conseguiu diferenciar entre três subtipos genéticos de SQTL.

Esta análise foi conduzida usando um nível de significância estatística de 5% (p = 0,05). IA: inteligência artificial; ECG: eletrocardiograma; SQTL: Síndrome do QT Longo.

Os artigos analisados se concentraram em três subtópicos da arritmologia cardíaca: Síndrome do QT Longo (SQTL), intervalo QT corrigido (QTc) e fibrilação atrial (FA). No estudo sobre SQTL, o objetivo foi avaliar a capacidade dos modelos de IA de detectar essa síndrome em indivíduos.^11^ Em relação à arritmia induzida por medicamentos, os pesquisadores buscaram avaliar a prevalência de arritmia por meio da análise automatizada do QTc em indivíduos submetidos a terapias específicas.^12^ A FA, por outro lado, apresentou o maior número de estudos, que visavam principalmente avaliar a precisão,^13 , 14^ viabilidade,^15 , 16^ confiabilidade,^14 , 15^ desempenho,^17^ eficiência,^18^ segurança,^18^ poder discriminativo,^19^ sensibilidade, especificidade e precisão^20^dos algoritmos e dispositivos associados utilizados para a detecção de episódios de FA. No entanto, outros estudos buscaram explicar o processo de tomada de decisão dos modelos de ML no diagnóstico de arritmias^21 - 23^Os tamanhos das amostras foram altamente variáveis. Todos os estudos utilizaram amostras de pacientes ou dados coletados de bancos de dados de instituições médicas associadas à pesquisa.

Analisando os resultados, a maioria dos resultados foi satisfatória e alinhada aos objetivos iniciais. Para modelos aplicáveis à Síndrome do QT Longo (SQTL), o algoritmo proposto distinguiu com sucesso as duas populações investigadas: indivíduos com SQTL e aqueles sem a síndrome.^11^ Em relação à detecção de FA usando algoritmos baseados em IA, os artigos relataram altas taxas de sensibilidade, especificidade, valor preditivo positivo (VPP), valor preditivo negativo (VPN), precisão e outras métricas de desempenho.^13 - 20 , 23^ Na avaliação de arritmias induzidas por terapia farmacológica, houve concordância entre os padrões de intervalo QTc derivados de ECGs convencionais e ECGs de dispositivos vestíveis.^12^ Quanto aos modelos algorítmicos explicativos para diagnóstico de arritmia, os resultados variaram: um estudo mostrou uma área sob a curva (AUC) relativamente moderada,^21^ enquanto outro demonstrou melhor desempenho.^22^

Em relação aos resultados e conclusões para o diagnóstico de SQTL usando IA, o ECG baseado em IA superou o método convencional em capacidade de distinção diagnóstica e foi capaz de diferenciar subtipos genéticos. Para o diagnóstico de FA usando IA, os algoritmos, modelos e dispositivos demonstraram alta sensibilidade na detecção de FA na última hora,^13^ maior sensibilidade e especificidade em comparação ao gerenciamento convencional e outros algoritmos comparativos,^15 , 17^ e maior precisão na detecção em diferentes posturas^18^e tempos.^19^ No contexto da monitorização de arritmias induzidas por fármacos, não houve variabilidade entre os modelos de IA e os ECGs convencionais.^12^ No entanto, modelos algorítmicos explicativos para diagnóstico de arritmia produziram resultados ambíguos: um apresentou limitação na detecção de FA durante o ritmo sinusal,^21^ enquanto outro correlacionou com sucesso as características dos traços de ECG com o diagnóstico de arritmia usando IA.^22^ Os desfechos, resultados e conclusões estão resumidos nas Tabelas 1 , 2 e 3 .

**Tabela 2:** – Síntese do Desenho, Resultados e Conclusões – Fibrilação Atrial

Autor, ano, (ref. Nº)	Projeto	Modelo de IA	Resultados	Conclusão
Wasserlauf et al., 2019 ^13^	Estudo de coorte prospectivo. Objetivo: Comparar a precisão de um dispositivo sensível à FA atrial com a gravação simultânea de um monitor cardíaco vestível. Amostra: Treinamento de IA com 7.500 dados de ECG; validação de coorte com 26 pacientes. Principais resultados: O dispositivo com sensor de ECG demonstrou alta sensibilidade para detectar FA na última hora em uma população ambulatorial.	Rede Neural Profunda	Dos 82 episódios detectados pelo monitor cardíaco implantável, o dispositivo sensível à FA identificou 80, alcançando uma sensibilidade de 97,5% por episódio.	Os resultados demonstram que o dispositivo vestível com o sensor de ECG, o aplicativo e o algoritmo investigativo são altamente sensíveis para detectar episódios de FA na última hora em uma população ambulatorial e para avaliar a duração da FA quando comparado com um MCI.
Huang et al., 2021 ^15^	Ensaio clínico randomizado. Objetivo: Avaliar a viabilidade e a confiabilidade de um dispositivo de ECG autoaplicável e sistema de monitoramento para detecção de FA. Amostra: 218 pacientes previamente submetidos à ablação, randomizados em dois grupos: grupo BT monitorado pelo algoritmo de IA e grupo TF monitorado por acompanhamento médico tradicional. Principais desfechos: A sensibilidade e a especificidade do algoritmo de IA foram superiores às da detecção automatizada de FA.	Algoritmo de IA	Viabilidade do algoritmo de IA no acompanhamento: 26.133 registros de ECG, com detecção de 12,6% de FA confirmada em revisão manual por cardiologistas, 14,8% pelo algoritmo automatizado de detecção de FA e 13,2% pelo algoritmo genérico de IA. O modelo de IA detectou mais recorrência de FA na FA paroxística após ablação (p = 0,0099), mas não na FA persistente (p = 0,7910). A sensibilidade e a especificidade do algoritmo genérico de IA para detecção de FA (94,4% e 98,5%) foram maiores do que as do algoritmo automático de detecção (90,7% e 96,2%).	O acompanhamento após ablação utilizando o algoritmo de IA leva à detecção mais frequente de recorrência de FA. O algoritmo de IA demonstrou maior precisão no diagnóstico de ECG e foi mais eficaz do que as estratégias tradicionais.
Jacobsen et al., 2020 ^17^	Estudo de coorte prospectivo. Objetivo: Avaliar o desempenho de um dispositivo médico vestível que utiliza tecnologia de fotopletismografia para detectar FA em pacientes hospitalizados com FA. Amostra: 102 pacientes internados em um hospital alemão com FA documentada. Principais resultados: O dispositivo demonstrou melhor capacidade de detecção de FA do que o monitoramento Holter tradicional.	Rede Neural Profunda	O dispositivo detectou episódios de FA no conjunto de dados com uma sensibilidade de 95,2% e especificidade de 92,5%.	A detecção de FA por um dispositivo médico vestível é uma abordagem viável e confiável.
Fu et al., 2021 ^18^	Estudo de coorte prospectivo. Objetivo: Avaliar a eficácia e a segurança da detecção de FA e fornecer um método confiável e não invasivo para o rastreamento e o tratamento da FA na prática diária. Tamanho da amostra: 114 pacientes. Principais resultados: ECG dinâmico vestível com algoritmo de IA pode detectar FA e analisar ritmos cardíacos em diferentes posturas e após exercícios.	Algoritmo de IA	O método detectou a ocorrência de FA na posição supina com acurácia de 96,4%, sensibilidade de 92,4%, especificidade de 100%, VPP de 100% e VPN de 93,8%. A detecção de FA na posição ortostática alcançou acurácia de 98,2%, sensibilidade de 100%, especificidade de 100%, VPP de 100% e VPN de 96,8%.	O ECG dinâmico variável pode detectar FA em ritmos cardíacos em diferentes posturas e após exercícios.
Noseworthye et al., 2022 ^19^	Ensaio clínico não randomizado. Objetivo: Avaliar o poder discriminativo do modelo de IA em adultos. Avaliar a eficácia da estratégia de rastreio de FA guiada por IA em comparação com o tratamento habitual. Amostra: 1.003 pacientes. Principais resultados: O modelo de IA detectou ritmos e duração da FA.	Algoritmo de IA	Cinquenta e quatro pacientes foram diagnosticados recentemente com FA com duração de 30 segundos ou mais e estratificados em grupos de baixo e alto risco. Um padrão semelhante foi observado nos grupos com FA com duração de 6 minutos ou mais e 24 horas ou mais. Alto risco foi associado a uma maior carga de FA (média de 4,97% para baixo risco; média de 20,32% para alto risco).	O dispositivo é capaz de detectar FA em vários momentos e estratificar o risco. Os resultados corroboram um programa de triagem guiado por IA, de baixo custo, altamente escalável e centrado no paciente.
Yang et al., 2022 ^21^	Estudo de coorte prospectivo. Objetivo: Propor uma abordagem de ML baseada em características que forneça resultados explicáveis e possa ser treinada em um banco de dados de tamanho viável. Amostra: Dados digitais de registros de ECG de 10 e 12 derivações de um hospital taiwanês. Principais resultados: O modelo foi capaz de isolar características temporais e espaciais das ondas P e prever padrões de FA.	Modelo de *Machine Learning*	Predição de FA usando o modelo de extração de onda P por machine learning: o modelo atingiu a maior AUC (0,64). O modelo apresentou especificidade moderada (0,71), mas baixa sensibilidade (0,47).	Os dados mostram poder limitado para as características propostas na detecção de pacientes com FA durante a RS. Com o processamento e a extração de características, é possível construir modelos para identificar FA potencial, mas o modelo ainda é impreciso.
Zhu et al., 2022 ^16^	Estudo de coorte prospectivo. Objetivo: Desenvolver, implementar e validar um algoritmo de detecção de FA baseado em fotopletismografia para *smartwatches* em pacientes com diagnóstico de FA. Amostra: 204 participantes. Desfecho principal: O algoritmo permitiu a detecção passiva de FA com base em fotopletismografia usando um dispositivo vestível.	Algoritmo de IA	O algoritmo detectou FA em 148 de 204 pacientes ao longo de 4 semanas. Sensibilidade: 87,8%; especificidade: 97,4%; poder de decisão (ritmo sinusal ou indeterminado): 67,8%.	O algoritmo demonstrou a viabilidade de detectar FA com precisão e estimar de forma não invasiva a carga da FA.
Jo et al.,2021 ^22^	Estudo retrospectivo. Objetivo: Desenvolver e validar um modelo de *deep learning* explicável (XDM) baseado em uma árvore de decisão apoiada por rede neural para classificação de arritmias. Amostra: Um conjunto de 72.740 registros de ECG de 42.880 pacientes de um hospital sul-coreano entre 2017 e 2020.	Modelo de *Deep Learning*	O escore F1 da validação externa do XDM foi: RSN (Ritmo Sinusal Normal): 0,990; FA ou Flutter Atrial: 0,955; Taquicardia Supraventricular: 0,777; Bloqueio AV Completo: 0,828; Ritmo do Marcapasso: 0,671; Acurácia agregada: 0,844. FA e flutter atrial foram fortemente correlacionados com características como a presença da onda P e irregularidade, enquanto o bloqueio AV completo mostrou forte correlação com a dissociação AV.	O modelo classifica com precisão arritmias em vários formatos de ECG usando conjuntos de dados de validação externa.
Jo et al., 2021 ^23^	Estudo retrospectivo. Objetivo: Propor um método para construir um XIA. Desenvolver e validar um modelo de *deep learning* explicável para detectar FA usando vários formatos de ECG. Amostra: 115.485 ECGs para desenvolvimento do modelo de IA; 12.914 ECGs para treinamento; 99.965 para validação externa. Principais resultados: O modelo superou a autointerpretação de ECG da máquina em todos os conjuntos de dados de validação. Também verificou o motivo (onda P e irregularidade) para sua conclusão.	Modelo de *Deep Learning*	O modelo de IA mostrou sensibilidade de 0,928, especificidade de 0,950, VPP de 0,615, VPN de 0,993 e precisão de 0,948 na detecção de FA.	Os resultados indicaram que a metodologia XIA poderia ser usada para descrever o raciocínio por trás da decisão do modelo de detectar FA com alto desempenho.
Chen et al., 2020 ^20^	Estudo de coorte prospectivo. Objetivo: Avaliar a sensibilidade, especificidade e precisão de um *smartwatch* com PPG e ECG que utiliza um algoritmo de IA para detecção de FA. Amostra: 401 pacientes entre maio e junho de 2019. Principais resultados: A sensibilidade, especificidade e precisão da pulseira equipada com algoritmos de PPG, ECG e IA demonstram desempenho satisfatório em curto prazo.	Algoritmo de IA	O dispositivo com PPG apresentou os seguintes resultados para detecção de FA: sensibilidade: 80,00%; especificidade: 96,81%; acurácia: 90,52%; VPP: 93,75%; e VPN: 89,01%.	O dispositivo apresentou boa sensibilidade, especificidade e precisão na determinação da presença de FA.
Väliaho et al., 2019 ^14^	Estudo de caso-controle. Objetivo: Avaliar a precisão de uma pulseira PPG comercialmente disponível na detecção de pulsos individuais na FA e avaliar a confiabilidade de dois algoritmos de detecção de FA comumente utilizados com base na PPG. Amostra: 213 pacientes no total, 106 para o grupo FA e 107 para o grupo RS (controle). Principais achados: A pulseira equipada com PPG, utilizando dois algoritmos de detecção de FA, foi capaz de diagnosticar FA com alta sensibilidade e especificidade.	Algoritmo de IA	A detecção de FA por PPG mostrou sensibilidade de 96,2% e especificidade de 98,1% com o algoritmo de evidência de FA e sensibilidade de 95,3% e especificidade de 98,1% com COSEn.	Pulseiras de fotopletismografia podem ajudar a detectar casos assintomáticos ou “silenciosos” de FA.

Todas as análises foram conduzidas usando um nível de significância estatística de 5% (p = 0,05). IA: inteligência artificial; ECG: eletrocardiograma; FA: fibrilação atrial; MCI: monitor cardíaco invasivo; VPP: valor preditivo positivo; VPN: valor preditivo negativo; XIA: modelo de IA explicável; PPG: fotopletismografia; AV: atrioventricular.

**Tabela 3:** – Síntese de Desenho, Resultados e Conclusões - Arritmia induzida por fármacos

Autor, ano, (ref. nº)	Maille et al., 2021^**12**^
Desenho do estudo	Estudo de coorte prospectivo. Objetivo: Comparar o QTc calculado usando este algoritmo em ECGs de derivação única de um smartwatch com o QTc medido em ECGs convencionais de 12 derivações em pacientes com COVID-19 em estágio inicial tratados com o regime HCQ-AZM. Amostra: um total de 85 pacientes; 76 em regime medicamentoso. Principais desfechos: Nas medições, houve concordância entre o padrão do intervalo QTc do ECG de 12 derivações e o QTc medido no SW-ECG correspondente.
Modelo IA	Rede neural convolucional.
Resultados	Concordância entre o intervalo QTc padrão de 12 derivações medido manualmente na derivação II ou V5 nos dias 0, 6 e 10 e o QTc IA medido no SW-ECG correspondente.
Conclusão	Há pouca diferença significativa entre os métodos de IA e ECG convencional para diagnosticar arritmia induzida por medicamentos.

Esta análise foi conduzida usando um nível de significância estatística de 5% (p = 0,05). IA: inteligência artificial; ECG: eletrocardiograma

## Discussão

Nesta revisão sistemática, os algoritmos automatizados para detecção de arritmia cobriram três apresentações principais: Síndrome do QT longo, outras anormalidades do intervalo QT e FA.

### Síndrome do QT longo

Na Síndrome do QT Longo, o estudo analisado nesta revisão conseguiu distinguir populações com uma acurácia de 82,2%, apresentando uma sensibilidade de 83,7% (VPP de 83,2%) e especificidade de 80,6% (VPN de 81,3%). Isso destaca que a aplicação de algoritmos de IA no diagnóstico dessa arritmia demonstra um grau satisfatório de acurácia, sensibilidade e especificidade.^11^Em relação às anormalidades do intervalo QT, o estudo incluído nesta revisão demonstrou concordância entre os resultados obtidos no ECG convencional de 12 derivações e no ECG com algoritmo de IA integrado a um dispositivo vestível equipado com tecnologia de fotopletismografia (PPG). Portanto, houve diferença mínima entre os achados do ECG convencional e os sugeridos pelo algoritmo de IA.^12^

Hermans et al.^24^ pesquisaram previamente características morfológicas que poderiam apoiar o diagnóstico de SQTL e outras variações do intervalo QT. Esses autores investigaram os valores agregados de marcadores morfológicos da onda T no início do estudo e em um modelo estendido. Concluíram que a morfologia da onda T tem um valor agregado na distinção de pacientes com SQTL de familiares com genótipo negativo. Essa característica morfológica pode explicar a precisão do trabalho de Bos et al.^11^ Em relação às anormalidades do intervalo QT, Prifti et al. ^25^relatam modelos de ML capazes de prever pacientes propensos a desenvolver prolongamento significativo do intervalo QT induzido por medicamentos.

Corroborando as contribuições de Simon et al.^26^nas alterações eletrofisiológicas do intervalo QT, a interpretabilidade se dá por meio da precisão preditiva. Métodos interpretáveis permitem uma inspeção completa das interações que levam a arritmias nessa classe, seja pela expressão de uma alteração genética ou por interações medicamentosas específicas. Essa interpretabilidade é útil na prevenção de complicações e deve ser considerada para integrar a modelagem preditiva em ferramentas de apoio à decisão clínica.

### Fibrilação atrial

Em relação à detecção geral de FA, caracterizada pela detecção de eventos de FA independentemente da duração, da posição do indivíduo ou de fatores morfológicos específicos do traçado eletrocardiográfico, os estudos demonstraram alto grau de acurácia, sensibilidade e especificidade. Wasserlauf et al.^13^e Chen et al.^20^demonstraram que seus algoritmos derivados de IA foram eficazes na detecção de eventos de FA. No primeiro caso, o design do sensor de ECG foi altamente sensível na detecção de episódios genéricos, enquanto no segundo caso, apresentou desempenho satisfatório na avaliação de curto prazo. Huang et al.^15^aprofundaram o desenho do estudo e descobriram que o algoritmo genérico de IA era mais preciso na detecção de FA em comparação ao diagnóstico humano e ao algoritmo específico para detecção de FA.

Na detecção de FA em diferentes posições corporais e após exercício, Fu et al.^18^destacaram alta precisão na detecção do evento tanto na posição supina quanto na ortostática, bem como após a realização de exercícios aeróbicos, com valores de precisão variando de 96,4% a 98,2%. Em relação à duração, Noseworthy et al.^19^ e Li et al.^16^ detectaram FA em avaliações diagnósticas com duração de 30 segundos, 6 minutos, 24 horas e até 4 semanas.

Uma fronteira da aplicação da IA em diversos campos é a compreensão dos resultados obtidos. No contexto da detecção de FA, é extremamente importante detectar fatores morfológicos que expliquem a saída do algoritmo. Para este fim, Yang et al.,^21^e Jo et al.^22 , 23^ modelaram e aplicaram algoritmos para isolar características-chave da FA. Yang et al.^21^tentaram quantificar mudanças temporais e espaciais no traçado de ECG para detecção de FA; no entanto, o processamento e a extração de características resultaram em um modelo impreciso. Jo et al.,^22^ por outro lado, demonstraram um modelo capaz de classificar a arritmia com precisão usando dados de validação externa. Em um estudo subsequente, eles conseguiram mostrar um modelo que superou a autointerpretação de um aparelho de ECG e identificou o raciocínio por trás da conclusão do achado.^23^ Portanto, estes últimos autores demonstraram sucesso tanto na detecção do evento de FA quanto na explicação das razões do resultado, com alta sensibilidade (92,8%) e especificidade (95%).^23^

A detecção de FA por meio de *smartwatch* é uma ferramenta presente nas publicações analisadas (4 de 13 publicações). Na comparação realizada por Wasserlauf et al.^13^ entre o monitor cardíaco invasivo (MCI) e o *smartwatch* , foi demonstrada alta sensibilidade para detecção de FA na última hora e na avaliação da duração do episódio arrítmico pelo dispositivo vestível. Em contraste, o MCI apresentou maior valor preditivo positivo para FA em episódios com duração superior a uma hora. Da mesma forma, Chen et al.^20^compararam o desempenho entre o *smartwatch* e o ECG de 12 derivações. Como resultado, encontraram boa sensibilidade (96,6%), especificidade (98%) e precisão (97,5%) na detecção de FA de curto prazo pelo dispositivo equipado com o algoritmo de IA.

Väliaho et al.^14^propuseram um estudo comparativo entre a detecção de ritmo de FA e ritmo sinusal utilizando smartwatches integrados a dois modelos de algoritmo de ML. Na detecção de FA, eles relataram alta sensibilidade (91,7%; VPP: 97,5%), enquanto na detecção de ritmo sinusal, relataram sensibilidade significativa (99,4%) e especificidade (98,1%). Os autores identificaram os casos de FA de início mais recente, mas declararam considerável confiabilidade na detecção de episódios de FA, independentemente de sua cronologia, com altas taxas de sensibilidade e especificidade.

### Arritmias induzidas por drogas

Por fim, com foco na detecção de arritmias induzidas por drogas, Maille et al.^12^compararam dados do intervalo QT obtidos de *smartwatches* e ECGs de 12 derivações. Neste estudo, os autores destacaram que, apesar da variabilidade no intervalo QT corrigido (QTc), houve concordância razoável entre os resultados gerados pela IA do *smartwatch* e os relatórios de ECGs convencionais. Com base nessa descoberta, os autores enfatizaram que o monitoramento de pacientes por meio de *smartwatches* oferece vantagens distintas, incluindo a capacidade de prever, detectar e prevenir arritmias potencialmente fatais.

Considerando os diversos resultados obtidos nesta revisão sistemática, fica evidente que algoritmos derivados de IA podem detectar padrões múltiplos, sutis e não lineares em um ECG. Consistente com a hipótese previamente confirmada por Attia et al.,^27^ essas redes demonstram maior sensibilidade na detecção de FA, mesmo em ECGs com ritmo sinusal normal. Os achados estão alinhados com os relatados na literatura médica atual, como a meta-análise de Feeny et al.^28^no diagnóstico de FA usando um algoritmo derivado de IA, que destaca altas taxas de sensibilidade (94%) e especificidade (96%).

Entretanto, apesar da diversidade de achados nesta revisão sistemática, há limitações metodológicas na indexação e disponibilização dos resultados nas bases de dados e nos desfechos dos dados da literatura elencados. As limitações relacionadas à metodologia deste artigo estão associadas à operacionalização por um único operador e ao espaço temporal da pesquisa. Em relação às bases de dados, a potencial indexação inadequada dos artigos pode suprimir resultados com critérios de inclusão positivos para a revisão. Em relação às limitações dos estudos elencados neste trabalho, o universo amostral e os delineamentos dos estudos são diversos e heterogêneos, contribuindo para vieses de seleção, confirmação e confusão decorrentes de predições oferecidas pela máquina que carregam vieses de seu modelo de treinamento.

Por outro lado, as tecnologias, em geral, podem levar à exclusão de grupos socialmente desfavorecidos, impedindo-os de acessar as funcionalidades oferecidas. Apesar da alta taxa de precisão na detecção de arritmias por dispositivos móveis vestíveis, estes requerem conexão com outro dispositivo, geralmente um *smartphone* , para maximizar sua usabilidade e funcionalidade. Por outro lado, essas tecnologias permitem o monitoramento de populações vulneráveis e indivíduos em regiões remotas que podem enfrentar disparidades no acesso a cuidados médicos.^29^

Assim, por ser uma área especializada e em rápida expansão, a ciência de dados aplicada à saúde requer estudos multicêntricos sobre os benefícios, as limitações metodológicas e os impactos clínicos e sociais das tecnologias no processo diagnóstico. Ao mesmo tempo, há um enorme escopo para a implementação de dispositivos baseados em algoritmos de IA na população para triagem, diagnóstico e subsequente tratamento precoce de arritmias potencialmente limitantes ou ameaçadoras à vida. A otimização desses processos é imperativa tanto do ponto de vista médico quanto socioeconômico, pois pode levar à redução de custos com hospitalizações e à melhoria da qualidade de vida. A aplicabilidade de recursos tecnológicos à prática da medicina baseada em evidências por meio do fornecimento contínuo de dados em larga escala para melhorar a precisão dos dispositivos eletrônicos de diagnóstico, algoritmos e conduta profissional.

## Conclusão

Com base nos resultados apresentados, a aplicação, validade e viabilidade de modelos de ML no diagnóstico de arritmias cardíacas representam uma área promissora e em rápido avanço. Esses modelos demonstraram alta precisão, sensibilidade e especificidade na detecção de anormalidades do ritmo cardíaco, particularmente FA. No entanto, sua generalização para outras populações permanece uma limitação, assim como o potencial para vieses introduzidos por tendências nos conjuntos de dados de treinamento. Além disso, a falta de estudos comparativos avaliando o desempenho da inteligência humana versus IA apresenta desafios para a validação completa de métodos algorítmicos como ferramentas confiáveis na prática médica de rotina. Assim, à medida que esse campo especializado continua a se expandir, estudos multicêntricos são essenciais para avaliar os benefícios, as restrições metodológicas e os impactos clínicos e sociais dessas tecnologias no processo diagnóstico.

## References

[1] World Health Organization (2021). Cardiovascular Diseases (CVDs).

[2] Tse G (2016). Mechanisms of Cardiac Arrhythmias. J Arrhythm.

[3] Jha CK (2024). Automated Cardiac Arrhythmia Detection Techniques: A Comprehensive Review for Prospective Approach. Comput Methods Biomech Biomed Engin.

[4] Pereira TMC, Sebastiao R, Conceicao RC, Sencadas V (2025). A Review on Intelligent Systems for ECG Analysis: From Flexible Sensing Technology to Machine Learning. IEEE J Biomed Health Inform.

[5] Hoffmann J, Mahmood S, Fogou PS, George N, Raha S, Safi S (2020). A Survey on Machine Learning Approaches to ECG Processing.

[6] Dorado-Díaz PI, Sampedro-Gómez J, Vicente-Palacios V, Sánchez PL (2019). Applications of Artificial Intelligence in Cardiology. The Future is Already Here. Rev Esp Cardiol.

[7] Shinde PP, Shah S (2018). A Review of Machine Learning and Deep Learning Applications.

[8] Lopez-Jimenez F, Attia Z, Arruda-Olson AM, Carter R, Chareonthaitawee P, Jouni H (2020). Artificial Intelligence in Cardiology: Present and Future. Mayo Clin Proc.

[9] Shameer K, Johnson KW, Glicksberg BS, Dudley JT, Sengupta PP (2018). Machine Learning in Cardiovascular Medicine: Are We There Yet?. Heart.

[10] Moher D, Liberati A, Tetzlaff J, Altman DG, PRISMA Group (2010). Preferred Reporting Items for Systematic Reviews and Meta-Analyses: The PRISMA Statement. Int J Surg.

[11] Bos JM, Attia ZI, Albert DE, Noseworthy PA, Friedman PA, Ackerman MJ (2021). Use of Artificial Intelligence and Deep Neural Networks in Evaluation of Patients with Electrocardiographically Concealed Long QT Syndrome From the Surface 12-Lead Electrocardiogram. JAMA Cardiol.

[12] Maille B, Wilkin M, Million M, Rességuier N, Franceschi F, Koutbi-Franceschi L (2021). Smartwatch Electrocardiogram and Artificial Intelligence for Assessing Cardiac-Rhythm Safety of Drug Therapy in the COVID-19 Pandemic. The QT-logs study. Int J Cardiol.

[13] Wasserlauf J, You C, Patel R, Valys A, Albert D, Passman R (2019). Smartwatch Performance for the Detection and Quantification of Atrial Fibrillation. Circ Arrhythm Electrophysiol.

[14] Väliaho ES, Kuoppa P, Lipponen JA, Martikainen TJ, Jäntti H, Rissanen TT (2019). Wrist Band Photoplethysmography in Detection of Individual Pulses in Atrial Fibrillation and Algorithm-Based Detection of Atrial Fibrillation. Europace.

[15] Huang S, Zhao T, Liu C, Qin A, Dong S, Yuan B (2021). Portable Device Improves the Detection of Atrial Fibrillation after Ablation. Int Heart J.

[16] Zhu L, Nathan V, Kuang J, Kim J, Avram R, Olgin J (2022). Atrial Fibrillation Detection and Atrial Fibrillation Burden Estimation via Wearables. IEEE J Biomed Health Inform.

[17] Jacobsen M, Dembek TA, Ziakos AP, Gholamipoor R, Kobbe G, Kollmann M (2020). Reliable Detection of Atrial Fibrillation with a Medical Wearable during Inpatient Conditions. Sensors.

[18] Fu W, Li R (2021). Diagnostic Performance of a Wearing Dynamic ECG Recorder for Atrial Fibrillation Screening: The HUAMI Heart Study. BMC Cardiovasc Disord.

[19] Noseworthy PA, Attia ZI, Behnken EM, Giblon RE, Bews KA, Liu S (2022). Artificial Intelligence-Guided Screening for Atrial Fibrillation Using Electrocardiogram during Sinus Rhythm: A Prospective Non-Randomised Interventional Trial. Lancet.

[20] Chen E, Jiang J, Su R, Gao M, Zhu S, Zhou J (2020). A New Smart Wristband Equipped with an Artificial Intelligence Algorithm to Detect Atrial Fibrillation. Heart Rhythm.

[21] Yang HW, Hsiao CY, Peng YQ, Lin TY, Tsai LW, Lin C (2022). Identification of Patients with Potential Atrial Fibrillation during Sinus Rhythm Using Isolated P Wave Characteristics from 12-Lead ECGs. J Pers Med.

[22] Jo YY, Kwon JM, Jeon KH, Cho YH, Shin JH, Lee YJ (2021). Detection and Classification of Arrhythmia Using an Explainable Deep Learning Model. J Electrocardiol.

[23] Jo YY, Cho Y, Lee SY, Kwon JM, Kim KH, Jeon KH (2021). Explainable Artificial Intelligence to Detect Atrial Fibrillation Using Electrocardiogram. Int J Cardiol.

[24] Hermans BJM, Stoks J, Bennis FC, Vink AS, Garde A, Wilde AAM (2018). Support Vector Machine-Based Assessment of the T-Wave Morphology Improves Long QT Syndrome Diagnosis. Europace.

[25] Prifti E, Fall A, Davogustto G, Pulini A, Denjoy I, Funck-Brentano C (2021). Deep Learning Analysis of Electrocardiogram for Risk Prediction of Drug-Induced Arrhythmias and Diagnosis of Long QT Syndrome. Eur Heart J.

[26] Simon ST, Trinkley KE, Malone DC, Rosenberg MA (2022). Interpretable Machine Learning Prediction of Drug-Induced QT Prolongation: Electronic Health Record Analysis. J Med Internet Res.

[27] Attia ZI, Harmon DM, Behr ER, Friedman PA (2021). Application of Artificial Intelligence to the Electrocardiogram. Eur Heart J.

[28] Feeny AK, Chung MK, Madabhushi A, Attia ZI, Cikes M, Firouznia M (2020). Artificial Intelligence and Machine Learning in Arrhythmias and Cardiac Electrophysiology. Circ Arrhythm Electrophysiol.

[29] Garikapati K, Turnbull S, Bennett RG, Campbell TG, Kanawati J, Wong MS (2022). The Role of Contemporary Wearable and Handheld Devices in the Diagnosis and Management of Cardiac Arrhythmias. Heart Lung Circ.

