# Supplementary material for: Aplicabilidade de Algoritmos de Machine Learning no Diagnóstico de Fibrilação Atrial e SQTL por Interpretação de Eletrocardiograma: Uma Revisão Sistemática
Source: Arq Bras Cardiol. 2025 Aug 12;122(8):e20240843. [Article in Portuguese] doi: 10.36660/abc.20240843 (PMC12677803; doi:10.36660/abc.20240843)
Supplement: Translation

# Translation: Applicability of Machine Learning Algorithms in Diagnosis of Atrial Fibrillation and LQTS by Electrocardiogram Interpretation: A Systematic Review

**DOI:** 10.36660/abc.20240843i

**Authors:** Paulo Cainan Guimarães do Nascimento, Matthews Silva Martins, Alex Cleber Improta-Caria, Roque Aras

**Affiliations:** 1 Brazil Universidade Federal da Bahia – Faculdade de Medicina da Bahia, Salvador, BA – Brazil; 2 Brazil Universidade Federal do Espírito Santo – Laboratório de Fisiologia Molecular e Inteligência Artificial, Departamento de Ciências Fisiológicas, Vitória, ES – Brazil; 3 Brazil Universidade de São Paulo – Laboratório de Bioquímica e Biologia Molecular do Exercício, Escola de Educação Física e Esporte, São Paulo, SP – Brazil

**Keywords:** Cardiac Arrhythmias, Artificial Intelligence, Machine Learning, Neural Network, Electrocardiography

## Abstract

**Background:**

Machine Learning (ML) is a type of algorithm that autonomously learns to recognize complex patterns. In the diagnostic context of cardiac arrhythmias, these algorithms have shown significant advancements due to their ability to provide automated interpretation and pattern recognition in electrocardiograms (ECGs).

**Objective:**

To analyze and identify the applicability, validity, and feasibility of ML algorithm models in the diagnostic process of cardiac arrhythmias through automated electrocardiogram interpretation.

**Methods:**

This systematic literature review was reported according to the PRISMA guidelines. The searches were conducted in the Cochrane Library, EMBASE, LILACS, and PubMed between February 2022 and November 2022. The study period encompasses articles published between 2017 and 2022.

**Results:**

The database search yielded 119 results, covering three subthemes: Long QT Syndrome (LQTS), corrected QT interval (QTc), and atrial fibrillation (AF). AF was the most prevalent theme. The sample sizes were quite variable. The outcomes were mostly satisfactory. In the diagnosis of LQTS using Artificial Intelligence (AI), the algorithm outperformed conventional methods in diagnostic distinction. In the evaluation of QTc, there was no difference between the AI-integrated ECG and the conventional ECG. In the diagnosis of AF, the algorithms, models, and devices demonstrated high sensitivity and specificity, along with greater accuracy.

**Conclusion:**

ML models in the diagnostic process of cardiac arrhythmias are feasible and rapidly developing. They demonstrate accuracy values between 96.4% and 98.2%, sensitivity between 92.8% and 99.4%, and specificity between 95% and 98.1%, particularly in the diagnosis of atrial fibrillation.

## Introduction

According to the World Health Organization, cardiovascular diseases (CVD) are the leading causes of death worldwide, accounting for 32%.^1^Among CVD, arrhythmias are the most common and are characterized by abnormalities in the generation or conduction of electrical impulses and occur due to abnormal heart function, resulting in irregular heart rhythms. The occurrence of arrhythmias may be indolent, but they can cause serious outcomes.^2 , 3^

The diagnostic approach for cardiac arrhythmias involves two main components: clinical history and electrocardiogram (ECG). The clinical history lists irregular heartbeats, dyspnea, fatigue, dizziness, and, when left untreated, a history of stroke and heart failure. The suspicion can be corroborated by changes in the cardiovascular physical examination through auscultation with a stethoscope and palpation of pulses. The diagnosis is confirmed with the recording of the ECG Holter for 24 hours and subsequent certification of irregular rhythms by a specialist physician. However, the conventional method of diagnosis by ECG Holter, in general, imposes limitations due to the lack of mechanical flexibility to the patient and the retrospective analysis method, without real-time monitoring and follow-up.^4 , 5^

However, Artificial Intelligence (AI) algorithms and their derivatives have emerged as a reliable method to identify and classify electrocardiographic patterns that may suggest abnormalities.^5^Dorado-Díaz et al.^6^refer to AI as a “field of computer science that attempts to mimic the human cognitive process, learning capacity, and knowledge storage”. In Health Sciences, AI algorithms are predominantly applied in prediction, recommendation, and diagnostic support based on Machine Learning (ML) and Deep Learning (DL) models. Conceptually, ML focuses on developing algorithms that enable computer systems to learn without explicit programming. In contrast, DL models derive their predictive capabilities from Artificial Neural Networks (ANNs) with multiple layers of information processing.^7 , 8^

In this context, ML algorithms are now capable of analyzing ECGs, providing automated interpretations, and predicting associated risks.^8^The literature highlights predictions derived from the optimization of procedural steps, such as signal processing, extraction of significant variables, and algorithm classification.^9^Despite the intrinsic computational complexity, there are several advances in systems that perform this role.

Therefore, AI tools can complement medical monitoring, from preventive to rehabilitation levels. The development of ML capable of conducting ECG analysis is essential in the early detection of abnormal cardiac conditions remotely and assisting the diagnostic process of arrhythmias and other health conditions.^5^Furthermore, long-term cardiac monitoring, recognition, and classification of arrhythmias based on the analysis of the ECG tracing can be a time-consuming and unnecessarily exhaustive process for the cardiologist. Thus, autonomic arrhythmia detection techniques are useful for such executions. In this scenario, computerized pattern recognition techniques will potentially help provide diagnosis and medical intervention whenever necessary.^3^

Considering the above, the study of AI and its derivative technologies in the healthcare field proves to be highly relevant. Therefore, this work aimed, through a systematic review of scientific literature, to explore the applicability, validity, and feasibility of ML models in the diagnostic process of cardiac arrhythmias. The study sought to identify methodological benefits and limitations, potential clinical and social impacts of these technologies, and their implications for the structure of medical education and practice.

## Methods

The study design is a systematic literature review conducted using the criteria established by the PRISMA method (Preferred Reporting Items for Systematic Reviews and Meta-Analyses), as detailed in the methodological guidelines for the preparation of systematic reviews and meta-analyses of randomized clinical trials provided by the Brazilian Ministry of Health.^10^

For the literature search on the intended topic, the following databases were used: Cochrane Library, Embase, LILACS (Latin American and Caribbean Health Sciences Literature), and PubMed-MEDLINE (Medical Literature Analysis and Retrieval System Online). The search terms were derived from the DeCS/MeSH (Health Sciences Descriptors/Medical Subject Headings) vocabulary, supplemented by entry terms. The descriptors included: arrhythmias; cardiac or cardiac arrhythmia; machine learning; unsupervised machine learning; supervised machine learning; deep learning; neural networks, computer or computer neural networks or computational neural network or computational neural networks; artificial intelligence or AI or computational intelligence; and electrocardiography or ECG or EKG. The search was conducted using the following configuration of descriptors and Boolean operators: ((cardiac arrhythmia) OR (arrhythmia) AND (machine learning)) OR (unsupervised machine learning)) OR (supervised machine learning)) OR (deep learning) OR (neural network) OR (artificial intelligence) AND (electrocardiography).

During the screening process, studies meeting the following inclusion criteria were considered: 1) research involving human subjects or data obtained from human databases; 2) publications in their final version in peer-reviewed scientific journals; 3) studies of the following types: case-control studies, cohort studies, clinical trials, case reports, and case series; 4) published between 2017-2022; 5) available in Portuguese or English. Exclusion criteria included: 1) publications in languages other than Portuguese or English; 2) studies with experimental descriptions lacking clinical application of the algorithmic model; 3) articles unrelated to the diagnosis of cardiac arrhythmias; 4) articles that do not apply AI models to the diagnosis of cardiac arrhythmias; 5) articles proposing methods for diagnosing cardiac arrhythmias using examinations other than ECG; 6) articles describing the use of AI for diagnosing conditions other than cardiac arrhythmias; 7) other types of publications, such as editorials, guidelines, books, systematic reviews, and meta-analyses; 8) duplicated results across the searched databases.

The screening process for search results and data extraction was based on the PRISMA method and carried out by a single operator. This process resulted in the creation of a database and a table model, enabling the organization of the retrieved data in configurations aligned with the employed method’s criteria serving as a precursor to future analyses. Using this methodology, the aim was to evaluate clinical studies addressing the use and applicability of ML algorithms in the diagnostic process of cardiac arrhythmias through the automated interpretation of ECGs.

## Results

The database search yielded a total of 119 results, including 38 from the Cochrane Library, 44 from Embase, none from LILACS, and 37 from PubMed. After applying the exclusion criteria, 98 publications were excluded for the following reasons: did not evaluate the detection of cardiac arrhythmias (83), did not apply AI algorithms for arrhythmia detection (5), addressed ongoing studies (3), were classified as other types of scientific texts (3), focused on an experimental model (1), described the diagnostic evaluation of arrhythmias using methods other than ECG (1), lacked a full-text version (1), and did not provide the full text in English or Portuguese (1). Consequently, 13 publications were deemed eligible for full and systematic review ( Figure 1 ).

**Figure 1:** – Flowchart of the Systematic Literature Review.

The 13 articles deemed eligible were included in the qualitative synthesis ( Table 1 ). The final search was conducted on November 30, 2022. Regarding the countries of origin of the included publications, they represented seven locations: the United States (4), China (3), South Korea (2), Germany (1), France (1), Taiwan (1), and Finland (1). In terms of study design, the articles comprised prospective cohort studies (7), retrospective cohort studies (2), case-control studies (2), and randomized clinical trials (2). All studies had received approval from the ethics committees of their respective institutions.

**Table 1:** – Synthesis of Design, Results, and Conclusions - LQTS

Author, year, (ref. Nº)	BOS, 2021^**11**^
Study design	Case-control study. Objective: Determine whether AI is capable of distinguishing patients with Long QT Syndrome from patients with a normal QTc using 12-lead ECG. Sample: 2.984 patients previously diagnosed with LQTS or discharged as healthy. Case-control diagnosis was performed using available 12-lead ECGs from 2.059 patients. Main outcome: AI was capable of distinguishing patients with LQTS, persistent QT prolongation, and genetic differentiation.
IA model	Deep Neural Network.
Results	AI was able to distinguish between the two populations (patients with LQTS and patients without LQTS). Accuracy: 82.2%; sensitivity: 83.7%; specificity: 80.6%; PPV: 83.2%; and NPV: 81.3%.
Conclusion	The AI-ECG outperformed conventional ECG in distinguishing patients with LQTS from those discharged without the diagnosis. Additionally, it could differentiate between three genetic subtypes of LQTS.

This analysis was conducted using a statistical significance level of 5% (p = 0.05). AI: artificial intelligence; ECG: electrocardiogram; LQTS: Long QT Syndrome.

The analyzed articles focused on three subtopics of cardiac arrhythmology: Long QT Syndrome (LQTS), corrected QT interval (QTc), and atrial fibrillation (AF). In the study on LQTS, the aim was to assess the ability of AI models to detect this syndrome in individuals.^11^Regarding drug-induced arrhythmia, the researchers sought to evaluate the prevalence of arrhythmia through automated analysis of QTc in individuals undergoing specific therapies.^12^AF, on the other hand, provided the highest number of studies, which primarily aimed to assess the accuracy,^13 , 14^feasibility,^15 , 16^reliability,^14 , 15^performance,^17^efficiency,^18^safety,^18^discriminative power,^19^sensitivity, specificity, and precision^20^of the algorithms and associated devices used to detect AF episodes. However, other studies aimed to explain the decision-making process of ML models in the diagnosis of arrhythmias.^21^The sample sizes were highly variable. All studies used patient samples or data collected from medical institution databases associated with the research.

Analyzing the outcomes, most results were satisfactory and aligned with the initial objectives. For models applicable to Long QT Syndrome (LQTS), the proposed algorithm successfully distinguished between the two populations under investigation: individuals with LQTS and those without it.^11^Regarding the detection of AF using AI-based algorithms, the articles reported high rates of sensitivity, specificity, positive predictive value (PPV), negative predictive value (NPV), accuracy, and other performance metrics.^13 - 20 , 23^In the assessment of arrhythmias induced by pharmacological therapy, there was concordance between the QTc interval patterns derived from conventional ECGs and wearable device ECGs.^12^As for explanatory algorithmic models for arrhythmia diagnostics, the outcomes varied: one study showed a relatively moderate area under the curve (AUC),^21^while another demonstrated better performance.^22^

Regarding the results and conclusions for diagnosing LQTS using AI, the AI-based ECG surpassed the conventional method in diagnostic distinction capabilities and was able to differentiate genetic subtypes. For the diagnosis of AF using AI, the algorithms, models, and devices demonstrated high sensitivity in detecting AF within the last hour,^13^higher sensitivity and specificity compared to conventional management and other comparative algorithms,^15 , 17^and greater accuracy in detection across different postures^18^and times.^19^In the context of monitoring drug-induced arrhythmias, there was no variability between AI models and conventional ECGs.^12^However, explanatory algorithmic models for arrhythmia diagnostics yielded ambiguous results: one exhibited limitation in detecting AF during sinus rhythm,^21^whereas another successfully correlated ECG trace characteristics with arrhythmia diagnosis using AI.^22^The outcomes, results, and conclusions are summarized in Tables 1 , 2 and 3 .

## Discussion

In this systematic review, the automated algorithms for arrhythmia detection covered three major presentations: Long QT Syndrome, other QT interval abnormalities, and AF.

### Long QT syndrome

In Long QT Syndrome, the study analyzed in this review was able to distinguish populations with an accuracy of 82.2%, presenting a sensitivity of 83.7% (PPV 83.2%) and specificity of 80.6% (NPV 81.3%). This highlights that the application of AI algorithms in diagnosing this arrhythmia demonstrates a satisfactory degree of accuracy, sensitivity, and specificity.^11^Regarding QT interval abnormalities, the study included in this review showed concordance between the results obtained from the conventional 12-lead ECG and the ECG with an AI algorithm integrated into a wearable device equipped with photoplethysmography (PPG) technology. Therefore, there was minimal difference between the findings of the conventional ECG and those suggested by the AI algorithm.^12^

Hermans et al.^24^previously researched morphological characteristics that could support the diagnosis of LQTS and other QT interval variations. These authors investigated the aggregated values of morphological markers of the T-wave at baseline and in an extended model. They concluded that T-wave morphology has an added value in distinguishing patients with LQTS from family members with a negative genotype. This morphological characteristic can explain the accuracy in the work of Bos et al.^11^Regarding QT interval abnormalities, Prifti et al.^25^report ML models capable of predicting patients prone to developing significant drug-induced QT prolongation.

Corroborating the contributions of Simon et al.^26^on electrophysiological changes in the QT interval, interpretability occurs through predictive accuracy. Interpretable methods allow for a thorough inspection of the interactions that lead to arrhythmias in this class, whether due to the expression of a genetic alteration or specific drug interactions. This interpretability is useful in preventing complications and should be considered for integrating predictive modeling into clinical decision-support tools.

### Atrial fibrillation

Regarding the general detection of AF, characterized by detecting AF events regardless of duration, the individual’s position, or specific morphological factors of the electrocardiographic trace, the studies showed a high degree of accuracy, sensitivity, and specificity. Wasserlauf et al.^13^and Erdong Chen et al.^20^demonstrated that their AI-derived algorithms were effective in detecting AF events. In the first case, the ECG sensor design was highly sensitive in detecting generic episodes, while in the second case, it showed satisfactory performance in short-term evaluation. Huang et al.^15^deepened their study design and found that the generic AI algorithm was more accurate in detecting AF compared to human diagnosis and the specific algorithm for detecting AF.

In the detection of AF in different body positions and after exercise, Fu et al.^18^highlighted high accuracy in detecting the event in both supine and orthostatic positions, as well as after performing aerobic exercise, with accuracy values ranging from 96.4% to 98.2%. Regarding duration, Noseworthy et al.^19^and Li et al.^16^detected AF in diagnostic assessments lasting 30 seconds, 6 minutes, 24 hours, and up to 4 weeks.

One frontier of AI application in various fields is the understanding of the results obtained. In the context of AF detection, it is extremely important to detect morphological factors that explain the algorithm’s output. To this end, Yang et al.^21^and Jo et al.^22 , 23^modeled and applied algorithms to isolate key features of AF. Yang et al.^21^attempted to quantify temporal and spatial changes in the ECG tracing for AF detection; however, the processing and feature extraction resulted in an imprecise model. Jo et al.,^22^on the other hand, demonstrated a model capable of accurately classifying the arrhythmia using external validation data. In a subsequent study, they were able to show a model that surpassed the self-interpretation of an ECG machine and identified the reasoning behind the conclusion of the finding.^23^Therefore, these latter authors demonstrated success both in detecting the AF event and in explaining the reasons for the result, with high sensitivity (92.8%) and specificity (95%).^23^

The detection of AF via smartwatch device is a tool present in the analyzed publications (4 of 13 publications). In the comparison made by Wasserlauf et al.^13^between the invasive cardiac monitor (ICM) and the smartwatch, high sensitivity for detecting AF in the last hour and in the assessment of the arrhythmic episode duration by the wearable device was demonstrated. In contrast, the ICM showed a higher positive predictive value for AF in episodes lasting longer than one hour. Similarly, Chen et al.^20^compared the performance between the smartwatch and the 12-lead ECG. As a result, they found good sensitivity (96.6%), specificity (98%), and accuracy (97.5%) in the short-term AF detection by the device equipped with the AI algorithm.

Väliaho et al.^14^proposed a comparative study between the detection of AF rhythm and sinus rhythm using smartwatches integrated with two ML algorithm models. In AF detection, they reported high sensitivity (91.7%; PPV: 97.5%), while in detecting sinus rhythm, they reported significant sensitivity (99.4%) and specificity (98.1%). The authors identified the most recent-onset AF cases but stated considerable reliability in detecting AF episodes regardless of their chronology, with high sensitivity and specificity rates.

### Drug-induced arrhythmias

Lastly, focusing on the detection of drug-induced arrhythmias, Maille et al.^12^compared QT interval data obtained from smartwatches and 12-lead ECGs. In this study, the authors highlighted that, despite variability in the corrected QT (QTc) interval, there was reasonable agreement between the outputs generated by the smartwatch’s AI and the reports from conventional ECGs. Based on this finding, the authors emphasized that patient monitoring through smartwatches offers distinct advantages, including the ability to predict, detect, and prevent potentially fatal arrhythmias.

Given the diverse results obtained in this systematic review, it is evident that AI-derived algorithms can detect multiple, subtle, and nonlinear patterns in an ECG. Consistent with the hypothesis previously confirmed by Attia et al.,^27^these networks demonstrate greater sensitivity in detecting AF, even in ECGs with normal sinus rhythm. The findings align with those reported in the current medical literature, such as the meta-analysis by Feeny et al.^28^on the diagnosis of AF using an AI-derived algorithm, which highlights high sensitivity (94%) and specificity (96%) rates.

However, despite the diversity of findings in this systematic review, there are methodological limitations in the indexing and provision of results in the databases and the outcomes of the listed literature data. The limitations related to the methodology of this article are associated with the operationalization by a single operator and the temporal space of research. Regarding the databases, the potential inadequate indexing of articles may suppress results with positive inclusion criteria for the review. Regarding the limitations of the studies listed in this work, the sample universe and study designs are diverse and heterogeneous, contributing to selection, confirmation, and confusion biases resulting from predictions offered by the machine that carry biases from its training model.

On the other hand, technologies, in general, can lead to the exclusion of socially disadvantaged groups, preventing them from accessing the functionalities offered. Despite the high accuracy rate in detecting arrhythmias by wearable mobile devices, these require connection to another device, usually a smartphone, to maximize their usability and functionality. On the other hand, these technologies allow the monitoring of vulnerable populations and individuals in remote regions who may face disparities in access to medical care.^29^

Thus, as it is a rapidly expanding and specialized area, data science applied to health requires multicenter studies on the benefits, methodological limitations, and clinical and social impacts of technologies in the diagnostic process. At the same time, there is enormous scope for implementing devices based on AI algorithms in the population for screening, diagnosis, and subsequent early treatment of potentially life-limiting or life-threatening arrhythmias. Optimizing these processes is imperative from both a medical and socioeconomic point of view, as they can lead to reduced costs for hospitalizations and improved quality of life. The applicability of technological resources to the practice of evidence-based medicine through the continuous provision of large-scale data to improve the accuracy of electronic diagnostic devices, algorithms, and professional conduct.

## Conclusion

Based on the presented results, the application, validity, and feasibility of ML models in diagnosing cardiac arrhythmias represent a promising and rapidly advancing area. These models have shown high accuracy, sensitivity, and specificity in detecting cardiac rhythm abnormalities, particularly AF. However, their generalizability to other populations remains a limitation, as does the potential for biases introduced by trends in the training datasets. Additionally, the lack of comparative studies assessing the performance of human intelligence versus AI presents challenges to fully validating algorithmic methods as reliable tools in routine medical practice. Thus, as this specialized field continues to expand, multicenter studies are essential to assess the benefits, methodological constraints, and clinical and societal impacts of these technologies in the diagnostic process.

**Table 2:** – Synthesis of Design, Results, and Conclusions – Atrial Fibrillation

Author, year, (ref. nº)	Design	AI model	Results	Conclusion
Wasserlauf et al., 2019 ^13^	Prospective cohort study. Objective: Compare the accuracy of an atrial AF-sensitive device with simultaneous recording from a wearable cardiac monitor. Sample: AI training with 7.500 ECG data; cohort validation with 26 patients. Main outcomes: The ECG sensor device demonstrated high sensitivity for detecting AF within the last hour in an ambulatory population.	Deep Neural Network	Of the 82 episodes detected by the implantable cardiac monitor, the AF-sensitive device identified 80, achieving a sensitivity of 97.5% per episode.	The results demonstrate that the wearable device with the ECG sensor, the application, and the investigative algorithm is highly sensitive for detecting AF episodes within the last hour in an ambulatory population and for assessing AF duration when compared with an ICM.
Huang et al., 2021 ^15^	Randomized clinical trial. Objective: To evaluate the feasibility and reliability of a self-applied ECG device and monitoring system for detecting AF. Sample: 218 patients previously submitted to ablation, randomized into two groups: BT group monitored by the AI algorithm and TF group monitored by traditional medical follow-up. Main outcomes: The sensitivity and specificity of the AI algorithm were higher than that of the automated AF detection.	AI Algorithm	Feasibility of the AI algorithm in follow-up: 26.133 ECG records, with detection of 12.6% of AF confirmed in manual review by cardiologists, 14.8% by the automated AF detection algorithm, and 13.2% by the generic AI algorithm. The AI model detected more AF recurrence in paroxysmal AF after ablation (p = 0.0099) but not in persistent AF (p = 0.7910). The sensitivity and specificity of the generic AI algorithm for detecting AF (94.4% and 98.5%) were higher than those of the automatic detection algorithm (90.7% and 96.2%).	Follow-up after ablation using the AI algorithm leads to more frequent detection of AF recurrence. The AI algorithm demonstrated improved accuracy of ECG diagnosis and was more effective than traditional strategies.
Jacobsen et al., 2020 ^17^	Prospective cohort study. Objective: To evaluate the performance of a wearable medical device employing photoplethysmography technology to detect AF in hospitalized patients with AF. Sample: 102 patients admitted to a German hospital with documented AF. Main outcomes: The device demonstrated better AF detection capability than traditional Holter monitoring.	Deep Neural Network	The device detected AF episodes in the dataset with a sensitivity of 95.2% and specificity of 92.5%.	AF detection by a wearable medical device is a feasible and reliable approach.
Fu et al., 2021 ^18^	Prospective cohort study. Objective: To assess the efficacy and safety of AF detection and provide a reliable, non-invasive method for the screening and management of AF in daily practice. Sample size: 114 patients. Main outcomes: Wearable dynamic ECG with AI algorithm can detect AF and analyze heart rhythms in different postures and after exercise.	AI algorithm	The method detected the occurrence of AF in the supine position with an accuracy of 96.4%, sensitivity of 92.4%, specificity of 100%, PPV of 100%, and NPV of 93.8%. The detection of AF in the orthostatic position achieved an accuracy of 98.2%, sensitivity of 100%, specificity of 100%, PPV of 100%, and NPV of 96.8%.	The dynamic variable ECG can detect AF in heart rhythms across different postures and after exercise.
Noseworthy et al., 2022 ^19^	Non-randomized clinical trial. Objective: To evaluate the discriminative power of the AI model in adults. Assess the effectiveness of the AI-guided AF screening strategy compared to usual care. Sample: 1.003 patients. Main outcomes: The AI model detected AF rhythms and duration.	AI algorithm	Fifty-four patients were newly diagnosed with AF lasting 30 seconds or more and stratified into low and high-risk groups. A similar pattern was observed in groups with AF lasting 6 minutes or more and 24 hours or more. High risk was associated with a higher AF burden (mean 4.97% for low risk; mean 20.32% for high risk).	The device is capable of detecting AF at various time points and stratifying risk. The results support a low-cost, massively scalable, patient-centered AI-guided screening program.
Yang et al., 2022 ^21^	Prospective cohort study. Objective: Proposition of a feature-based ML approach that provides explainable results and can be trained on a feasibly sized database. Sample: Digital data from 10- and 12-lead ECG recordings from a Taiwanese hospital. Main outcomes: The model was able to isolate temporal and spatial features of P waves and predict AF patterns.	Machine Learning model	AF prediction using machine learning P-wave extraction model: the model achieved the highest AUC (0.64). The model showed moderate specificity (0.71) but low sensitivity (0.47).	The data show limited power for the proposed features in detecting patients with AF during RS. With feature processing and extraction, it is possible to build models to identify potential AF, but the model is still imprecise.
Zhu et al., 2022 ^16^	Prospective cohort study. Objective: Develop, implement, and validate a photoplethysmography-based AF detection algorithm for smartwatches in patients diagnosed with AF. Sample: 204 participants. Main outcome: The algorithm enabled passive detection of AF based on photoplethysmography using a wearable device.	AI algorithm	The algorithm detected AF in 148 out of 204 patients over 4 weeks. Sensitivity: 87.8%; specificity: 97.4%; decision power (whether sinus rhythm or indeterminate): 67.8%.	The algorithm demonstrated the feasibility of accurately detecting AF and non-invasively estimating the burden of AF.
Jo et al., 2021 ^22^	Retrospective study. Objective: To develop and validate an explainable deep learning model (XDM) based on a neural network-backed decision tree (NBET) for arrhythmia classification. Sample: A set of 72.740 ECG records from 42.880 patients from a South Korean hospital between 2017 and 2020.	Deep Learning model	The F1 score of the external validation of the XDM was: NSR (Normal Sinus Rhythm): 0.990; AF or Atrial Flutter: 0.955; Supraventricular Tachycardia: 0.777; Complete AV Block: 0.828; Pacemaker Rhythm: 0.671; Aggregate accuracy: 0.844. AF and atrial flutter were strongly correlated with characteristics such as the presence of the P wave and irregularity, while complete AV block showed a strong correlation with AV dissociation.	The model accurately classifies arrhythmias in various ECG formats using external validation datasets.
Jo et al., 2021 ^23^	Retrospective study. Objective: Propose a method to construct an explainable AI (XAI) model. Develop and validate an explainable deep learning model for detecting AF using various ECG formats. Sample: 115.485 ECGs for AI model development; 12.914 ECGs for training; 99.965 for external validation. Main outcomes: The model outperformed the machine’s self-interpretation of ECG in all validation datasets. It also verified the reason (P wave and irregularity) for its conclusion.	Deep Learning model	The AI model showed a sensitivity of 0.928, specificity of 0.950, PPV of 0.615, NPV of 0.993, and accuracy of 0.948 in detecting AF.	The results indicated that the XAI methodology could be used to describe the reasoning behind the model’s decision to detect AF with high performance.
Chen et al., 2020 ^20^	Prospective cohort study. Objective: Evaluate the sensitivity, specificity, and precision of a smartwatch with PPG and ECG that uses an AI algorithm for AF detection. Sample: 401 patients between May and June 2019. Main outcomes: The sensitivity, specificity, and accuracy of the wristband equipped with PPG, ECG, and AI algorithms demonstrate satisfactory performance in the short term.	AI algorithm	The device with PPG showed the following results for AF detection: sensitivity: 80.00%; specificity: 96.81%; accuracy: 90.52%; PPV: 93.75%; and NPV: 89.01%.	The device provided good sensitivity, specificity, and accuracy in determining the presence of AF.
Väliaho et al., 2019 ^14^	Case-control study. Objective: Evaluate the accuracy of a commercially available PPG wristband in detecting individual pulses in AF and assess the reliability of two commonly used AF detection algorithms based on PPG. Sample: 213 total patients, 106 for the FA group and 107 for the SR group (control). Main findings: The wristband equipped with PPG, using two AF detection algorithms, was able to diagnose AF with high sensitivity and specificity.	AI algorithm	The detection of AF by PPG showed a sensitivity of 96.2% and specificity of 98.1% with the AF evidence algorithm and a sensitivity of 95.3% and specificity of 98.1% with COSEn.	Photoplethysmography wristbands can help detect asymptomatic or “silent” cases of AF.

All analyses were conducted using a statistical significance level of 5% (p = 0.05). AI: artificial intelligence; ECG: electrocardiogram; FA: atrial fibrillation; MCI: invasive heart monitor; VPP: positive predictive value; VPN: negative predictive value; XAI: explainable AI; PPG: photoplethysmography; AV: atrioventricular.

**Table 3:** – Synthesis of Design, Results, and Conclusions - Drug-induced arrhythmia

Author, year, (ref. nº)	Maille et al., 2021^**12**^
Study design	Prospective cohort study. Objective: Compare the QTc calculated using this algorithm on single-lead ECGs from a smartwatch with the QTc measured on conventional 12-lead ECGs in early-stage COVID-19 patients treated with the HCQ-AZM regimen. Sample: a total of 85 patients; 76 under medication regimen. Main outcomes: In the measurements, there was agreement between the QTc interval standard of the 12-lead ECG and the QTc measured in the corresponding SW-ECG.
IA model	Convolutional neural network.
Results	Agreement between the standard 12-lead QTc interval measured manually in the lead II or V5 on days 0, 6, and 10 and the AI-QTc measured in the corresponding SW-ECG.
Conclusion	There is little significant difference between the AI and conventional ECG methods for diagnosing drug-induced arrhythmia.

This analysis was conducted using a statistical significance level of 5% (p = 0.05). AI: artificial intelligence; ECG: electrocardiogram.
